# Supplementary material for: Incidence and survival in laryngeal and lung cancers in Finland and Sweden through a half century
Source: PLoS One. 2022 May 27;17(5):e0268922. doi: 10.1371/journal.pone.0268922 (PMC9140270; doi:10.1371/journal.pone.0268922)
Supplement: Attachment — Submitted filename: 22larynxFISEreponse to Rewiers.docx [file pone.0268922.s001.docx]

2. We note that the grant information you provided in the ‘Funding Information’ and ‘Financial Disclosure’ sections do not match.

Changed

4. We note that you have included the phrase “data not shown” in your manuscript. Unfortunately, this does not meet our data sharing requirements.

>>> We revised this in the end of the second paragraph of Results, p.5, giving the actual incidence rates.

**Comments to the Author**

5. Review Comments to the Author

Please use the space provided to explain your answers to the questions above. You may also include additional comments for the author, including concerns about dual publication, research ethics, or publication ethics. (Please upload your review as an attachment if it exceeds 20,000 characters)

Reviewer #1: I would like to mention the follwoing comments:

1- The comparability of data in two countries needs to be explained.

>>> We added text to the second sentence in Methods, p.4. Note that this theme is highlighted also in the last paragraph of Introduction, p. 4.

2- The potential confounders have not been considered.
>>> Text added (to strengths/weaknesses paragraph), p. 7.

3- The affecting factors on incidence have not been discussed.

>>> The overwhelming influence of smoking is discussed in the above section and throughout, including the first paragraph if Introduction.

4- Were APC (Age, Period, Cohort) applied for time trends?

>>> We added text to methods, p.4, birth cohort analysis results to p. 5, and the new Fig. 2, and Discussion first paragraphs, p. 6.

5- The applied statistical package?

>>> Explained in Methods, paragraph 2, p. 4.

Good Luck

Reviewer #2: This study addressed the incidence and survival of laryngeal and lung cancers in Finland and Sweden through a half-century. The results are useful for public health. However, the following concerns should be addressed.
Abstract
1. A part of the abstract section should be dedicated to the methodology of the study. Furthermore, only the main results, regarding the objective of the study (incidence and survival), should be reported and other findings can be presented in the main text. The conclusion should be meaty and concise as well.

>>> Abstract was modified accordingly.

Introduction
2. The introduction is too long. Some parts can be removed such as the second half of the first paragraph.

>>> The second half was deleted. Treatment should not be deleted in survival studies.

3. The introduction section should include an overview of what is known on the topic, an explanation of what is unknown, and finally a description of what will be addressed by the study.

>>> These points are discussed in paragraphs 2 and 3 of Introduction.

Methods
4. The statistical software used for analysis as well as the significance level should be specified.

>>> Explained in Methods, paragraph 2, p. 4.
Results
5. The total number of lung and laryngeal cases should be reported in the first paragraph of the main results.

>>> Done, p. 5.

Conclusion
6. Since the analyses are performed on ecologic data, no individual data on smoking status is available. Therefore, linking fluctuations of the incidence rate of lung and laryngeal cancers to the prevalence of smoking in this study does not make sense.

>>> When smoking is by far the overwhelming contributioning factor it is playing a role at the population level (see first paragraph of Introduction, p. 3 and the new text on Discussion, p. 7: correlation coefficient 0.91!)
